# 2109. HHV-6 and EBV Reactivation after Allogeneic Hematopoietic Cell Transplantation in the Era of Letermovir for CMV Prophylaxis: A Retrospective Cohort Study

**DOI:** 10.1093/ofid/ofac492.1730

**Published:** 2022-12-15

**Authors:** Eleftheria Kampouri, Elizabeth M Krantz, Danniel Zamora, Louise E Kimball, Erika Kiem, Erika A Lovas, Rachel Blazevic, Terry L Stevens-Ayers, Meei-Li Huang, Ueda Oshima Masumi, Keith R Jerome, Danielle M Zerr, Michael J Boeckh, Joshua A Hill

**Affiliations:** Fred Hutchinson Cancer Center, Seattle, WA; Fred Hutch Cancer Center, Seattle, Washington; Fred Hutchinson Cancer Center, Seattle, WA; Fred Hutchinson Cancer Center, Seattle, WA; Fred Hutchinson Cancer Center, Seattle, WA; Fred Hutchinson Cancer Center, Seattle, WA; Fred Hutchinson Cancer Research Center, Seattle, WA; Fred Hutchinson Cancer Center, Seattle, WA; University of Washington, Seattle, Washington; Fred Hutchinson Cancer Center, Seattle, WA; Fred Hutchinson Cancer Center, Seattle, WA; University of Washington; Seattle Children's Research Institute, Seattle, Washington; Fred Hutchinson Cancer Center, Seattle, WA; Fred Hutchinson Cancer Center; University of Washington, Seattle, Washington

## Abstract

**Background:**

Letermovir (LTV) for CMV prophylaxis in allogeneic hematopoietic cell transplant (HCT) recipients has decreased utilization of broad-spectrum antivirals for CMV, which may result in more frequent reactivation of herpesviruses not targeted by LTV. We hypothesized that the cumulative incidence of HHV-6 and EBV reactivation, and associated diseases, within 100 days after HCT would increase following the introduction of LTV.

**Methods:**

We conducted a retrospective study of CMV-seropositive adults who received an allogeneic HCT at our center prior to (5/2015 – 9/2018) and after (10/2018 – 12/2021) routine use of LTV. We reviewed medical records for antiviral use, viral testing, and virus-associated end-organ disease within days 0-100 after HCT. Testing was performed at the discretion of healthcare providers or according to treatment protocols. We computed cumulative incidence estimates treating death and subsequent HCT as competing risks.

**Results:**

We identified 403 patients prior to and 378 patients after routine use of LTV. Characteristics were similar between cohorts except for more frequent use of post-transplant cyclophosphamide for GVHD prophylaxis in the LTV cohort. Broad-spectrum antiviral use, as estimated by available data, was 68% pre-LTV versus 23% post-LTV. The incidence of tests for and detection of HHV-6 were similar in both cohorts (**Figure 1**). Two patients in each cohort (0.5%) developed proven or probable HHV-6 encephalitis. Median peak plasma HHV-6 viral load was nearly 1 log_10_ higher in the LTV cohort (**Figure 2**). The incidence of tests for and detection of EBV was higher in the LTV cohort, but the proportion of patients with ≥ 1 positive test among tested patients was similar (14% pre-LTV vs 20% post-LTV); of note, EBV testing in both cohorts was mainly protocol driven (57% and 50% among tested patients pre- and post-LTV, respectively). There were no cases of PTLD by day 100 in either cohort.

**Figure:**
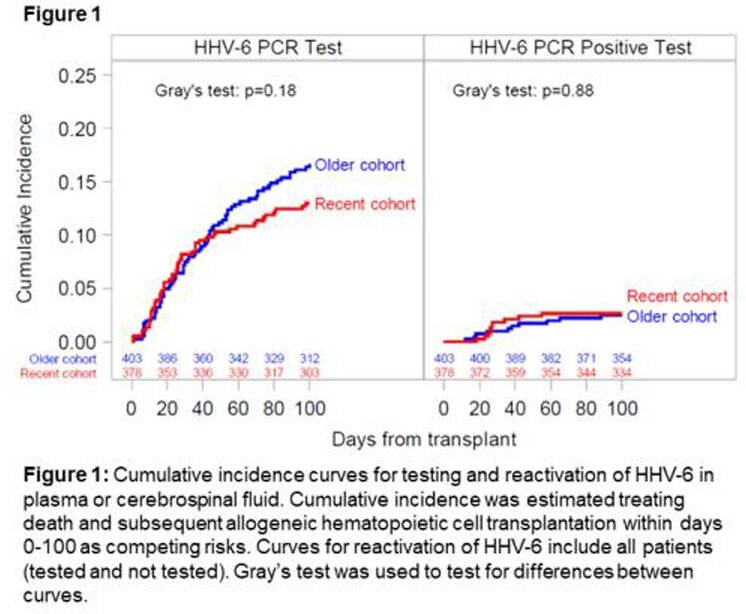

**Figure:**
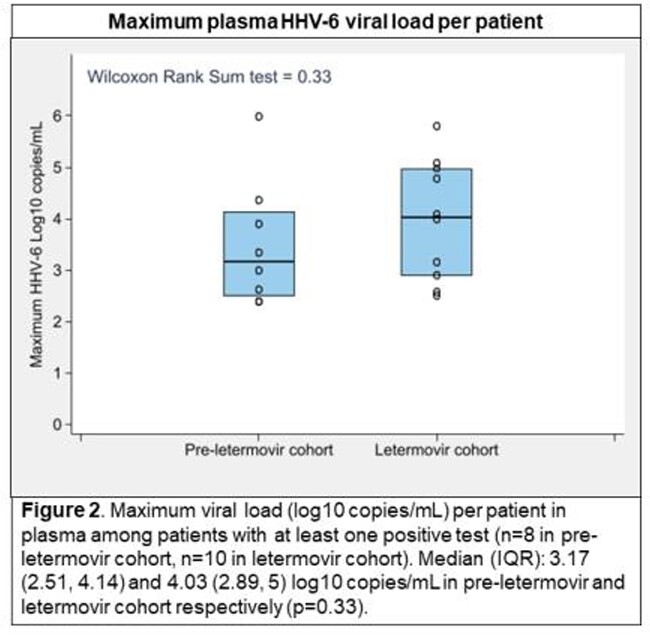

**Conclusion:**

Despite a 66% decrease in broad-spectrum antiviral use after the introduction of LTV prophylaxis in CMV-seropositive allogeneic HCT recipients, there was no evidence for increased HHV-6 or EBV reactivation and associated end-organ diseases. Whether the higher peak HHV-6 viral load in the LTV cohort has clinical implications requires further study.

**Disclosures:**

**Danielle M. Zerr, MD MPH**, AlloVir: Advisor/Consultant **Michael J. Boeckh, MD PhD**, Allovir: Advisor/Consultant|Amazon: Grant/Research Support|Ansun Biopharma: Grant/Research Support|EvrysBio: Advisor/Consultant|Gates Ventures: Grant/Research Support|Gilead Sciences: Advisor/Consultant|Gilead Sciences: Grant/Research Support|GlaxoSmithKline: Advisor/Consultant|GlaxoSmithKline: Grant/Research Support|Helocyte: Advisor/Consultant|Janssen: Advisor/Consultant|Janssen: Grant/Research Support|Kyorin Pharmaceuticals: Advisor/Consultant|Merck: Advisor/Consultant|Merck: Grant/Research Support|Moderna: Advisor/Consultant|Moderna: Grant/Research Support|Regeneron: Grant/Research Support|ReViral: Advisor/Consultant|Symbio: Advisor/Consultant|Takeda: Grant/Research Support|Vir Biotechnology: Advisor/Consultant|Vir Biotechnology: Grant/Research Support **Joshua A. Hill, MD**, Allovir: Advisor/Consultant|Allovir: Grant/Research Support|Covance/CSL: Advisor/Consultant|CRISPR: Advisor/Consultant|Deverra: Grant/Research Support|Gilead: Grant/Research Support|Karius: Advisor/Consultant|Karius: Grant/Research Support|Merck: Grant/Research Support|Octapharma: Advisor/Consultant|OptumHealth: Advisor/Consultant|Oxford Immunotec: Grant/Research Support|Pfizer: Advisor/Consultant|Symbio: Advisor/Consultant|Takeda: Advisor/Consultant.

